# Wound Conforming Matrix Containing Purified Homogenate of Dermal Collagen Promotes Healing of Diabetic Neuropathic Foot Ulcers: Comparative Analysis Versus Standard of Care

**DOI:** 10.1089/wound.2019.1024

**Published:** 2019-12-18

**Authors:** Lois A. Chandler, Oscar M. Alvarez, Peter A. Blume, Paul J. Kim, Robert S. Kirsner, John C. Lantis, William A. Marston

**Affiliations:** ^1^ Olaregen Therapeutix, Inc., New York, New York.; ^2^ Vascular and Wound Care Center, University Hospital, Newark, New Jersey.; ^3^ Departments of Surgery, Anesthesia, and Cardiology, Yale School of Medicine, New Haven, Connecticut.; ^4^ Department of Plastic Surgery and Orthopedic Surgery, University of Texas Southwestern Medical Center, Dallas, Texas.; ^5^ Department of Dermatology and Cutaneous Surgery, University of Miami Miller School of Medicine, Miami, Florida.; ^6^ Division of Vascular and Endovascular Surgery, Mount Sinai St. Luke's–West Hospitals, New York, New York.; ^7^ Division of Vascular Surgery, Department of Surgery, University of North Carolina School of Medicine, Chapel Hill, North Carolina.

**Keywords:** diabetic foot ulcer, collagen, platelet activation, randomized controlled trial, wound healing rate

## Abstract

**Objective:** To compare outcomes of diabetic foot ulcers (DFUs) treated with a collagen Wound Conforming Matrix (WCM) or standard of care (SOC).

**Approach:** WCM, a highly purified homogenate of 2.6% fibrillar bovine dermal collagen that conforms to the wound surface, was evaluated in comparison to daily saline-moistened gauze dressing changes (SOC) as part of a retrospective subset analysis of a randomized controlled trial in DFU. Following a 2-week run-in period during which patients received SOC, patients whose wounds did not reduce in area by >30% during run-in were randomly assigned to receive WCM (one or two applications) or SOC.

**Results:** Statistically significant acceleration of early healing rates was observed following a single application of WCM with weekly outer dressing changes compared with daily saline-moistened gauze dressing changes (SOC). Over a 4-week period, 50% of patients receiving a single application of WCM achieved ≥75% reduction in wound area compared with 13% for SOC. WCM appeared to be safe and well tolerated, with no adverse events related to treatment and no evidence of an immunologic reaction to bovine collagen.

**Innovation:** WCM is unique in its intimate contact with the wound bed and its ability to progress a wound toward healing with a single application.

**Conclusion:** WCM is a treatment modality to accelerate DFU healing rates, with the potential to reduce the likelihood of infection and other complications, and cost of care.

**Figure:**
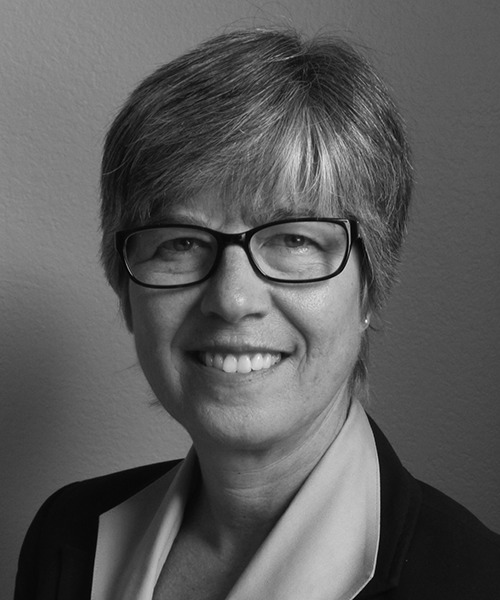
Lois A. Chandler, PhD

## Introduction

Diabetic foot ulcers (DFUs) are a common complication of diabetes mellitus (DM), affecting 19–34% of patients during their lifetime, and are associated with significant morbidity, mortality, and health care costs.^1^ The financial burden of DFU on public and private payers accounts for approximately one-third of the total cost of diabetic care and is estimated to range from $9 to $13 billion.^2,3^ We describe results from a retrospective, exploratory subset analysis of a randomized, controlled, multicenter clinical trial in DFU,^4^ comparing the efficacy and safety of one (*n* = 10) or two (*n* = 16) applications of a type I bovine fibrillar collagen-based Wound Conforming Matrix (WCM) versus daily saline-moistened gauze dressing changes (SOC, *n* = 15).

## Clinical Problem Addressed

DFUs are a common complication of DM and are associated with significant morbidity, mortality, and health care costs.^1^ The majority of DFU in this study were located on the plantar surface of the foot. Plantar shear stress is a major causative agent in the development of DFU and in the poor healing of DFU.^5^ Although the principles of DFU care are established, including offloading to redistribute pressure away from an ulcer, sharp debridement, moisture balance, and infection control,^3,6^ there remain gaps between desired and realized healing outcomes.^7^ New treatment modalities are needed to promote healing of chronic DFU and accelerate DFU healing rates, with potential to reduce the likelihood of infection and other complications, and cost of care.

## Materials and Methods

Design of the U.S. clinical study followed the guidelines put forth by the U.S. Food and Drug Administration in their June 2006 guidance document entitled, “Chronic Cutaneous Ulcer and Burn Wounds—Developing Products for Treatment,”^8^ and the study was approved at each participating site by the institutional review board and conducted in compliance with the Declaration of Helsinki (as amended in 2000). The study period was from November 2007 (first patient randomized) to October 2009 (last patient visit). The primary endpoint of the study was the incidence of complete ulcer closure by week 12 (complete epithelialization of the ulcer with no drainage, with closure confirmed at the next 2 weekly visits). After closure, wound assessments continued for 12 weeks to assess durability. Secondary endpoints included changes in ulcer area from baseline at weekly intervals, and safety. Enrollment criteria included patients with type 1 or type 2 DM over age 18 years with a lower extremity Wagner Classification Grade 1 ulcer present for at least 6 weeks. To limit the study to refractory DFUs, exclusion criteria included a decrease in ulcer area of >30% with SOC during a 2-week run-in period. For complete study criteria, refer to https://clinicaltrials.gov/ct2/show/NCT00493051 website.

Following qualification and written informed consent, patients underwent sharp debridement of the ulcer, a 4 mm wound biopsy for culture, clinical ulcer assessment, and ulcer photograph on day-14 to start a screening 2-week run-in period with offloading and daily saline-moistened gauze dressing changes (SOC). Sharp debridement was conducted to remove all necrotic soft tissue, hyperkeratotic wound margins, bacterial burden, cellular debris, sinus tracts, fistulae, undermined borders, and callus to produce viable wound margins and a bleeding wound bed. If either beta-hemolytic streptococci (in any amount) or total bacterial load of 1 × 10^6^ colony-forming unit per gram of tissue was present in the screening biopsy, the patient was given a single 7-day course of topical antibiotics and then re-debrided and biopsied for quantitative culture. A second biopsy and culture result exceeding the limits above resulted in exclusion from the study. During the run-in period, all patients were provided supplies for daily at-home SOC, consisting of application of saline-moistened gauze directly to the wound, coverage of the moist dressing with nonadherent Telfa^®^ (Cardinal Health, Dublin, OH), securing the dressings with a stretch bandage, and offloading with a special orthopedic shoe (DH Walker^®^; Ossur, Coconut Creek, FL).

On day-3, repeat clinical ulcer assessment was performed and qualified patients were randomly assigned 1:1:1 to the treatment groups that are the subject of this retrospective, exploratory subset analysis: (1) SOC, (2) WCM, single application on day 1, and (3) WCM, application on days 1 and 29. All patients wore the orthopedic offloading shoe throughout the study period.

The day 1 visit consisted of clinical assessment of the ulcer site, ulcer photograph, and sharp debridement. WCM was applied to wounds by site personnel at a volume of 0.1 mL/cm^2^ of post-debridement wound area and in the presence of a small influx of blood, and the wound was covered with nonadherent Telfa and left undisturbed for 1 week. This procedure was repeated at week 4 for those assigned to the WCM two-application group. Patients assigned to SOC continued with daily at-home saline-moistened gauze dressing changes. All patients were seen and assessed weekly in the clinic until ulcer closure or week 12. Sharp debridement was performed at screening, before treatment, and as clinically necessary at the weekly clinic visits. Ulcer closure was defined as complete epithelialization of the ulcer with no drainage, with closure confirmed at the next 2 weekly visits. Rate of healing was determined using weekly wound areas as measured by tracing calibrated photos using the NIH ImageJ software. Statistical comparisons between treatment groups were performed using Fisher's exact two-sided test and the Wilcoxon–Mann–Whitney test.

Activation of human platelets by WCM was evaluated *in vitro* in collaboration with Piedmont Research Center (Morrisville, NC). Fixed amounts of human platelet concentrate (ZenBio, Research Triangle Park, NC) were incubated with phosphate-buffered saline (negative control), bovine thrombin (positive control; King Pharmaceuticals, Bristol, TN), or increasing amounts of WCM and incubated for 20 min at 37°C. After further incubation for 24 h, samples were centrifuged and supernatants were assayed for human PDGF A/B by ELISA (R&D Systems, Minneapolis, MN). The PDGF A/B isoform was selected for measurement based on favorable assay sensitivity.

## Results

In the clinical study, a total of 37 patients were randomly assigned to receive WCM (one or two applications), and 19 patients were randomly assigned to receive daily saline-moistened gauze dressing changes (SOC). As reported by Blume *et al.*,^4^ comparison of baseline wound areas determined by acetate tracing and photograph analysis revealed systematically inaccurate (larger) measurements with acetate tracings. Therefore, the analyses reported herein were conducted using photo-based measurements, excluding patients with protocol violations and/or with wounds that achieved wound area reduction of >30% during the run-in period.

The original clinical study protocol was designed to exclude patients with baseline wound areas of <1.5 cm^2^. However, even small DFU can be difficult to heal, and the potential complications from small DFU can be as devastating and costly as large DFU. Therefore, wounds with baseline areas of <1.5 cm^2^ (WCM mean duration 9 months, SOC mean duration 7 months) are included in the analyses reported herein, resulting in 26 WCM-treated patients and 15 SOC-treated patients for analysis.

Demographics for the subset patient population are presented in Table 1. No significant difference between WCM and SOC was found for any baseline variable. Sharp debridement was performed at screening, on the day 1 visit, and as clinically necessary thereafter during the weekly clinic visits for both WCM and SOC. Frequency of debridement at clinic visits was 62% for WCM and 68% for SOC, a nonsignificant difference.

**Table 1.** Patient demographics

Variable	WCM^a^ (*N* = 26)	SOC (*N* = 15)
Age (years), mean ± SD	55 ± 12	57 ± 13
Gender, *n* (%)		
Male	19 (73)	11 (73)
Female	7 (27)	4 (27)
Baseline ulcer size (cm^2^), mean ± SD	2.0 ± 1.2	2.7 ± 1.7
Ulcer duration (months), mean ± SD	12.1 ± 11.8	12.9 ± 13.1
Ulcer location, *n* (%)		
Plantar	22 (85)	13 (87)
Lateral surface	3 (11)	0 (0)
Dorsal	1 (4)	2 (13)

^a^ One application, *n* = 10; Two applications, *n* = 16.

SD, standard deviation; SOC, standard of care; WCM, Wound Conforming Matrix.

**Figure 1.**
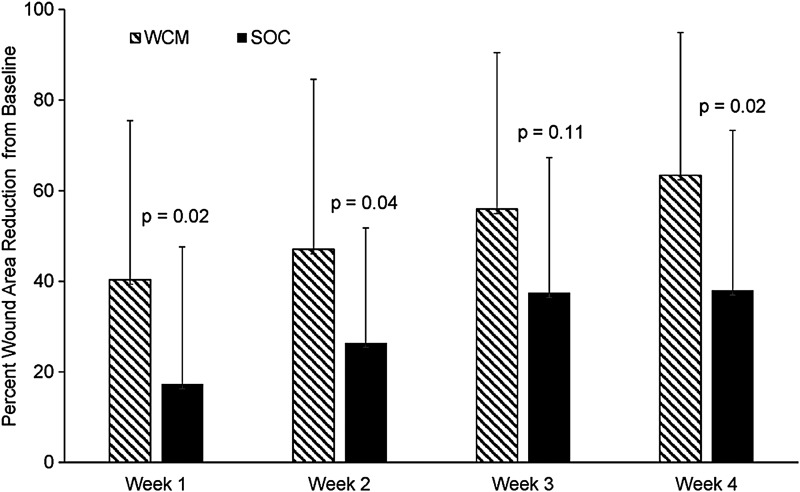
Cumulative percent wound area reduction from baseline through week 4 (mean ± SD). WCM (*n* = 26), SOC (*n* = 15). WCM versus SOC: week 1 (*p* = 0.02); week 2 (*p* = 0.04); week 3 (*p* = 0.11); week 4 (*p* = 0.02). WCM and SOC compared by the Wilcoxon–Mann–Whitney test. SD, standard deviation; SOC, standard of care; WCM, Wound Conforming Matrix.

The cumulative mean area reductions over the first 4 weeks, at which time all WCM patients had received a single application, are presented in Fig. 1. The data demonstrate a greater reduction in wound area between WCM (*n* = 26) and SOC (*n* = 15) at all 4 weeks, with statistically significant differences at weeks 1 (*p* = 0.02), 2 (*p* = 0.04), and 4 (*p* = 0.02). At week 4, the average wound area reduction with WCM was 63% compared with 38% for SOC. There was no significant difference in the percent wound area reduction at week 4 for patients assigned to receive one versus two applications of WCM (69% vs. 61%, respectively), and 13 (87%) of the patients receiving a second application of WCM received ≤0.1 mL at the second application, reflecting the dramatic reduction in wound area by week 4.

**Figure 2.**
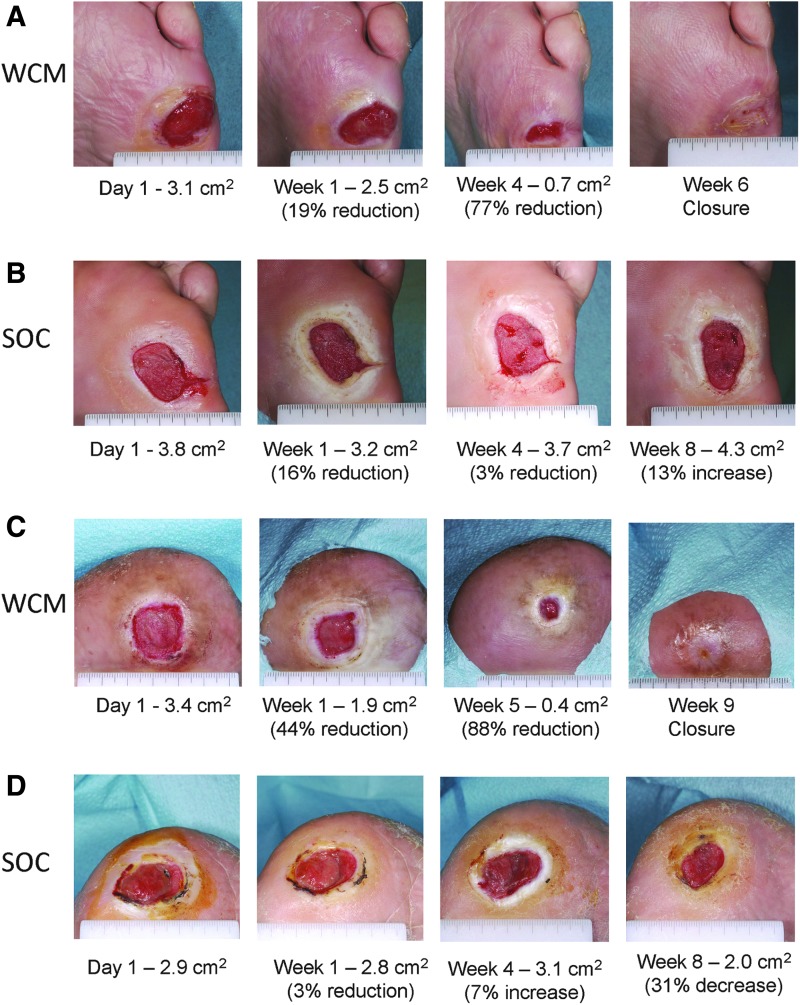
**(A)** A 59-year-old male with DFU of 3 months duration on plantar surface of left foot. Application of WCM on day 1 and week 4. **(B)** A 51-year-old female with DFU of 4 months duration on plantar surface of left foot. Daily saline-moistened gauze dressing changes. **(C)** A 42-year-old female with DFU of 13 months duration on plantar surface of right foot. Application of WCM on day 1. **(D)** A 63-year-old male with DFU of 51 months duration on plantar surface of right foot. Daily saline-moistened gauze dressing changes.

The 12-week complete closure incidences were 42% (11/26) for WCM and 27% (4/15) for SOC, a nonsignificant difference. Of the 11 wounds that closed following treatment with WCM, 1 reopened after 4 weeks, and 1 reopened after 12 weeks. More patients were progressing to closure with a single application of WCM compared with daily SOC, and WCM demonstrated acceleration of healing after application. By 2 weeks after a single application of WCM, 38% (10/26) of patients achieved ≥75% wound area reduction, compared with 7% (1/15) for SOC, a significant difference (*p* = 0.03). At 4 weeks after a single application of WCM, 50% (13/26) of patients achieved ≥75% wound area reduction, compared with 13% (2/15) for SOC, a significant difference (*p* = 0.02). Wound photos from two WCM-treated patients and two SOC-treated patients, with plantar location and approximate size-matched baseline areas, are presented in Fig. 2.

Safety of topically applied WCM was assessed by adverse events, clinical laboratory measurements, vital signs, concomitant medications, physical examination findings, and serum antibody concentrations to collagen. There were no application site infections in any of the WCM- or SOC-treated patients during the study period. There were no obvious or concerning safety trends in the hematology, chemistry, urine analysis, and other laboratory parameters for the WCM-treated patients compared with SOC. Safety of WCM was also assessed by monitoring levels of collagen antibodies at screening and week 5. Week 5 antibody titers to bovine collagen were positive for one patient treated with WCM and one patient treated with SOC. Both these patients also had positive collagen antibody titers at screening. Pre-existing antibodies to collagen did not affect treatment response, with wound closure achieved at week 5 for the WCM-treated patient. All other patients tested negative for collagen antibodies pre- and post-treatment. Therefore, there was no detectable serum antibody response to treatment with WCM.

**Figure 3.**
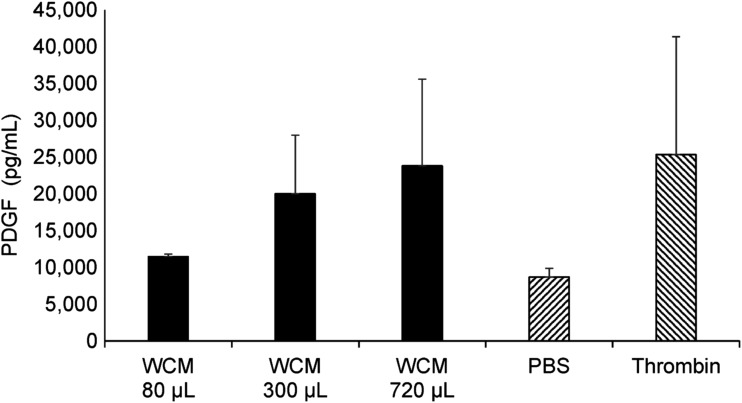
WCM-activated PDGF release. Fixed amounts of human platelet concentrate were incubated with PBS (negative control), bovine thrombin (positive control), or 80–720 μL of WCM and incubated for 20 min at 37°C. After 24 h, samples were assayed for human PDGF A/B by ELISA (mean ± SD). ELISA, enzyme-linked immunosorbent assay; PBS, phosphate-buffered saline; PDGF, platelet-derived growth factor.

The subset analyses reported herein were conducted due to the unexpected finding that WCM, originally intended as a control group, resulted in accelerated healing and wound closure incidences compared with daily saline-moistened gauze dressing changes (SOC).^4^ This finding prompted further investigation into the potential mechanism of action of WCM, in addition to its ability to function as a structural scaffold in dermal wound healing, as was demonstrated in preclinical studies.^9^ We confirmed *in vitro* that exposure of human platelets to WCM results in platelet activation and dose-dependent release of platelet-derived growth factor (PDGF), an essential mediator of the wound healing cascade (Fig. 3).

## Discussion

Although the basic principles of DFU care are established, including offloading to redistribute pressure away from an ulcer, sharp debridement, dressings to promote moisture balance, and infection control,^3,6^ there remain gaps between desired and realized healing outcomes with current standard of care strategies. There are numerous topical products available for use in the early management of DFU, including wet-to-dry dressings, hydrogels, hydrocolloids, alginates, and foam dressings. DFUs are heterogeneous, and unfortunately, DFU and other chronic wounds often fail to respond to conservative standard of care, requiring more advanced treatment options including cellular- and tissue-based products.^10^ Wound area reduction of >50% after 4 weeks of treatment has been found to be predictive of wound closure outcomes for DFU, venous leg ulcers, and chronic wounds overall.^11–13^ In this retrospective exploratory analysis, a single application of WCM accelerated healing with an average wound area reduction of 63% at 4 weeks.

The majority of DFU in this study were located on the plantar surface of the foot. Plantar shear stress is a major causative agent in the development and poor healing of DFU.^5^ A single application of WCM, with weekly outer dressing changes, demonstrated statistically significant acceleration of healing within 1 week of application, and persisting for 4 weeks, compared with daily saline-moistened gauze dressing changes (SOC). The average wound duration of the WCM and SOC treatment groups was ∼1 year. It is feasible that the daily dressing changes associated with SOC disrupted the healing process compared with once weekly outer dressing changes for WCM-treated patients. Debridement was performed on all wounds at the day 1 treatment visit, and frequency of debridement at subsequent visits was similar for WCM and SOC. Furthermore, there were no treatment site infections during the study period in any of the WCM- or SOC-treated patients.

The WCM manufacturing process was specifically designed to generate a highly purified homogenate of type I collagen that could conform to the wound bed while retaining the three-dimensional scaffold structure of native fibrillar collagen. The ability of WCM to activate human platelets is confirmation of its fibrillar structure.^14^ Endogenous PDGF plays an important role in each phase of the wound healing process, including stimulation of chemotactic recruitment and proliferation of cells involved in wound repair.^15^ Preclinical studies using animal models of wound healing have demonstrated that WCM provides a structural scaffold for cellular migration and proliferation,^9^ and this function combined with platelet activation may account for the acceleration of healing observed following a single application of WCM. In future clinical studies, it will be of value to measure PDGF levels within the wound following treatment with WCM to identify potential correlations between healing response and platelet activation by WCM.

The clinical trial protocol was limited to Wagner Grade 1 DFU and limited treatment with WCM to one or two applications, with the second application being applied at week 4. In the real-world setting, weekly application of WCM may be beneficial to maintain accelerated DFU healing rates and to increase closure incidences. Furthermore, the strict inclusion/exclusion criteria required by the clinical study protocol may not accurately represent the general population of patients with DFU. In addition to daily wet-to-dry dressings, the SOC employed in this exploratory analysis, there are other topical products available for standard of care of DFU, including hydrogels, hydrocolloids, alginates, and foam dressings. Detailed information regarding prior treatments was insufficient to determine specific product classes that failed to heal the study wounds before study enrollment. A larger clinical data set based on real-world experience with WCM is needed to establish the efficacy of WCM in healing DFU that have failed to heal with other specific classes of product. The potential benefits of the early acceleration of healing observed with WCM include reduced risk of infection, hospitalization and amputation, decreased health care costs, and positive impact on patients’ quality of life.

## Innovation

WCM has unique properties that promote healing of DFU, which is critical for predicting healing, reducing the risk of complications, and decreasing the cost of care. Manufactured as a sterile homogenate of purified fibrillar collagen, WCM conforms to the entire wound surface without the manipulations required with sheet-based products (*e.g.*, trimming and fixation). The acceleration of healing observed following a single application of WCM is attributed to its three-dimensional fibrillar structure, which provides a structural scaffold for repair cells, including those potentially stimulated by PDGF release from platelets activated by interaction with WCM.

**Key Findings**

- A single application of WCM achieves statistically significant acceleration of DFU healing rates compared with daily saline-moistened gauze dressing changes.
- A single application of WCM achieves >50% wound area reduction at 4 weeks, a benchmark found to be predictive for healing of chronic wounds.
- WCMs acceleration of healing is attributed to its three-dimensional fibrillar collagen structure and wound conforming properties, providing a structural matrix for repair cell migration and proliferation.

## About the Authors

**Lois A. Chandler, PhD,** is the Inventor of WCM and serves as Scientific Consultant for Olaregen Therapeutix. **Oscar M. Alvarez, PhD,** is Program Director of the Vascular and Wound Care Center and Director of Clinical Research at University Hospital, Newark, New Jersey. **Peter A. Blume, DPM, FACFAS,** is Director and President at Affiliated Foot and Ankle Surgeons, New Haven, Connecticut, and Assistant Clinical Professor at Yale University School of Medicine Departments of Orthopaedics and Surgery. **Paul J. Kim, DPM, MS,** is Medical Director of the Wound Program and Professor in the Department of Plastic Surgery and Orthopedic Surgery at the University of Texas Southwestern Medical Center, Dallas, Texas. **Robert S. Kirsner, MD, PhD,** is Chairman and Professor at the Dr. Phillip Frost Department of Dermatology and Cutaneous Surgery, University of Miami Miller School of Medicine. **John C. Lantis, MD,** is Chief of Vascular and Endovascular Surgery, Mt. Sinai West and St. Luke's Hospitals, New York, New York. **William A. Marston, MD,** is Professor in the Division of Vascular Surgery at the University of North Carolina School of Medicine and Medical Director of the UNC Limb Salvage/Wound Healing Center.

## Abbreviations and Acronyms

DFU: diabetic foot ulcer
DM: diabetes mellitus
ELISA: enzyme-linked immunosorbent assay
PBS: phosphate-buffered saline
PDGF: platelet-derived growth factor
SD: standard deviation
SOC: standard of care
WCM: Wound Conforming Matrix
